# Effect of nano h-BN particles on growth regularity and tribological behavior of PEO composite ceramic coating of ZL109 alloy

**DOI:** 10.1038/s41598-022-04768-0

**Published:** 2022-01-19

**Authors:** Xinhe Zhu, Jingguo Fu, Dengqing Ma, Chunsheng Ma, Yunyang Fu, Zhuokai Zhang

**Affiliations:** 1 Dalian Maritime University, Marine Engineering College, Liaoning Dalian, China; 2 Shenzhen Research Institute of Dalian Maritime University, Shenzhen, Guangzhou China

**Keywords:** Materials science, Mechanical engineering

## Abstract

A composite ceramic coating containing h-BN particles was prepared on the ZL109 alloy via plasma electrolytic oxidation. The h-BN particles were modified by Polyethylene glycol to improve the dispersibility. The results revealed that the h-BN particles in the electrolyte were inertly incorporated into the coating. Meanwhile, the incorporation of h-BN particles can reduce the porosity and slightly increase the roughness of the composite ceramic coating. Furthermore, the growth rate of the coating and the conversion of γ-Al_2_O_3_ and α-Al_2_O_3_ were promoted by the incorporation of h-BN particles via the change of the current. In addition, due to the presence of h-BN particles, the composite ceramic coating had a lower friction coefficient and a lower wear rate under dry sliding condition.

## Introduction

Aluminum alloys and their composite materials are widely used in aerospace, automobile and other industries because of their superior properties, such as high specific strength and low density^1,2^. However, due to low hardness and high chemical activity, their application in some specific conditions, such as high temperature, frictional and corrosive environment, are limited^3,4^. Some scholars have proposed several surface technologies including anodization^5^, chemical conversion coating^6^, laser finishing^7^, electroplating^8^, PVD^9^, and PEO^10,11^ to extend their applications. Among these technologies, the utilization of PEO to form a high-efficient, high wear and corrosion resistance ceramic film on the surface of materials is a promising surface treatment technology^4^. Furthermore, the properties^12,13^, growing mechanism^14,15^ and various parameters affecting the performance of the PEO coating^16,17^ are also investigated by some researchers.

In recent years, the preparation of PEO composite ceramic coating with some specific properties by directly introducing some functional particles into the electrolyte has become a new research focus. Because of its relatively simple operation, it is also called one-step method. For example, particles such as GO^18^, ZrO_2_^19^, CeO_2_^20^ are added into the electrolyte to prepare the corrosion resistant composite ceramic coating. BaTiO_3_^21,22^ and ZrO_2_^23^ are introduced to the electrolyte to prepare the dielectric insulating composite ceramic coating. α-Al_2_O_3_^24^, Fe_2_O_3_^25^, ZnSO_4_^26^ and SiC^27^ are added to the electrolyte to prepare the thermal protection and thermal control composite ceramic coating. CuO^28^, Tb_4_O_7_^29,30^, ZnO^31^ and K_4_[Fe(CN)_6_]^32^ are introduced to the electrolyte to prepare the catalytic composite ceramic coating. In terms of the anti-friction and wear-resistant composite ceramic coating, the added materials are mainly diamond^33^, CNTs^34^, PTFE^35^, graphite^36,37^ and MoS_2_^38^. Considering that the h-BN particles have high chemical stability, high thermal conductivity, layered structural characteristic and self-lubricating ability, Ao et al.^39^ have tried to directly introduce h-BN particles in the electrolyte to form composite ceramic coating on Ti–6Al–4V alloy by PEO method. The results show that better tribological behavior are observed.

However, hard aluminum alloys are also widely used in the wear and friction field, such as middle and high speed diesel engine pistons. Consequently, it is necessary to study the anti-friction effects of h-BN particles on PEO coatings on aluminum alloys, which are rarely investigated. Besides this, the existing researches on PEO composite ceramic coating mainly focus on improving the performance of the coatings by changing particle types and concentration^23,34,35,37,39^, while the growth regularity and mechanism are barely mentioned. Therefore, in this work, the h-BN particles are added as a self-lubricating material to the electrolyte to prepare a self-lubricating composite ceramic coating on aluminum alloy ZL109 which is the material of engine piston. The growth regularity, phase composition and tribological properties of the composite ceramic coating are investigated. Furthermore, the deposition mechanisms are also clarified herein.

## Experimental

The ZL109 alloy specimens were cut into blocks with a size of 50 mm × 10 mm × 10 mm. The chemical compositions of the alloy were 84.8 wt.% Al, 12.4 wt.% Si, 0.8 wt.% Cu, 1.05 wt.% Mg and 0.95 wt.% Ni. Prior to PEO processing, the surfaces of the specimens were sequentially grounded by 600#, 800#, 1200# abrasive papers, and then polished to obtain a lower roughness surface (Ra 0.12). Finally, ultrasonic cleaning with acetone and ethanol were carried out for 5 min, respectively, followed by drying in warm air.

During the experiment, the constant voltage mode was selected on the power supply^40,41^. Other major electrical parameters, including positive voltage, negative voltage, frequency, duty cycle and pulse ratio were set to + 420 V, − 120 V, 500 Hz, 20% and 1:1, respectively. An alkaline aqueous electrolyte was prepared from a solution of Na_2_SiO_3_ (8 g/L), Na_2_WO_4_·2H_2_O (5 g/L), EDTA-2Na (2.5 g/L), KOH (2 g/L). Finally, 2.5 g/L of h-BN particles (99.8%, < 150 nm), which were optimized in the previous work were added to the electrolyte^42^. A nonionic surfactant (Polyethylene glycol (Aladdin)) was used to disperse the particles and improve their dispersibility in the electrolyte. Its mass ratio to the h-BN particles is 2:1^43^. During the PEO treatment, the electrolyte temperature was controlled at 30 °C–40 °C by a cooling system. The specimen and a stainless-steel sink were used as the anode and cathode, respectively.

In order to better analyze the effects of h-BN particles on the evolution process and the growth regularity of PEO coating, experiments were carried out at different times. For ease of presentation, the specimens were named A1, B1, C1, D1 and A2, B2, C2, D2, respectively, as shown in Table 1.

**Table 1:** Experimental measurement scheme.

Time/(min)	10	20	30	40
Without h-BN	A1	B1	C1	D1
With h-BN	A2	B2	C2	D2

The roughness tester TR200 was used to examine the surface roughness of the coatings. Each surface was measured 5 times and the average value was used as the result. The morphology of surface, the cross-section and the elements composition in a specific area of the sample were observed and analyzed by a scanning electron microscopy (TESCAN VEGA3) equipped with an energy dispersive spectrometer (OXFORD). Commercial software Image J was adopted to measure the pore characteristics. X-ray diffraction (XRD, EMPYREAN) was performed using a diffractometer to determine the coating phase composition of the coating.

The tribological behaviors of the specimens were evaluated by a ball-on-disc reciprocating tribotester with a Si_3_N_4_ ball (Diameter 4 mm, HRC 68). The test was carried out under dry sliding condition with a normal load of 8N for 30 min. The rotating speed of the motor is 400 rpm. The reciprocating amplitude is 5 mm. Each tribotest was done at least two times, until the friction coefficient was almost the same in two times. As an important evaluation criteria of tribological property, the wear scars on the coating surface were observed using a three-dimensional surface profiler (OLYMPUS, OLLE5-74S14/7E-2). The wear rate was utilized to evaluate the anti-wear performance and was calculated by the following equations^44^:

1 $$ W_{v} = \frac{Lh}{{6b}}\left( {3h^{2} + 4b^{2} } \right) $$

2 $$ R = \frac{{W_{v} }}{N \times S} $$

where $W_{v}$ is the wear volume (mm^3^), $L$, $h$ and $b$ are the length (mm), depth (mm) and width (mm) of wear scar, respectively. $R$ is the wear rate (mm^3^·N^-1^·m^-1^), $N$ is the normal load (N), and $S$ is the total sliding distance (m).

## Results and discussion

### Electrical parameter-time response characteristic

**Figure 1:**
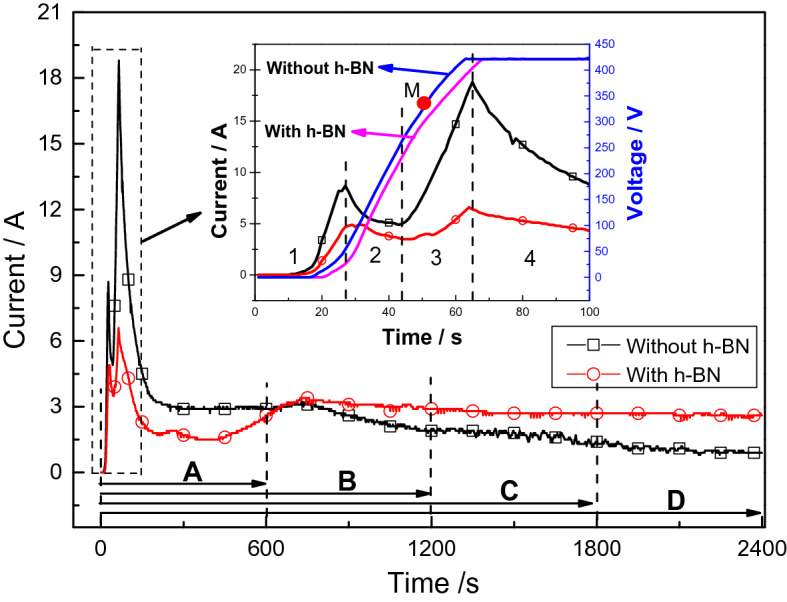
Electrical parameter-time response characteristics during the PEO process.

Figure 1 shows the characteristics of the electrical parameters in different oxidation periods during the PEO treatment. It can be seen that the addition of h-BN particles in the electrolyte has a significant effect on the evolution of current. When there is no h-BN particles in the electrolyte, the oxidation current experiences two rapid rises and falls during the first 10 min, with the maximum current reaching 19.5 A at about 65 s. After that, the current continues to decline slowly and eventually stabilizes at 0.9 A. There is only a little spark on the sample surface at the end of PEO process. When the h-BN particles are introduced into the electrolyte, similar trend of current variation is observed in the first 10 min. However, the current value is much lower than that without particles, and the rates of rises and falls are also significantly reduced. Moreover, it is interesting to note that the current experiences a short period of gradually increase after the rapid fall, which is quite different from that without h-BN particles. The period lasts for 10 min and then the current is maintained at 2.6 A until the end of the PEO treatment. But the partial discharge phenomenon is still severe at the end, which may be attribute to the severe static discharge around the bubbles on the surface^35^.

In addition, it can also be observed from Fig. 1 that the voltage response rate of the electrolyte with h-BN particles is smaller than that of the electrolyte without particles. As represented by point M in Fig. 1, the breakdown voltage of the electrolyte without h-BN particles is 327 V. For the electrolyte with h-BN particles, the breakdown voltage cannot be well confirmed due to the occlusion of sight caused by the milky solution during the experiment. However, it is known from the current profiles that the current response time of the two electrolytes is the same in the first 10 min. Therefore, it can be inferred that the breakdown voltage of the electrolytes with and without h-BN particles occurs at the same time. Accordingly, the breakdown voltage of the electrolyte with h-BN particles is found to be about 310 V, which is also lower than that without particles. The reason may be related to the properties of the h-BN particles. The h-BN particles can be negatively charged after being modified by polyethylene glycol^43^. When being introduced to the electrolyte, the negatively charged particles are more easily adsorbed onto the surface of the anode under the action of electric field. Therefore, the anodic oxide film on the surface is formed at a faster rate, resulting in a lower breakdown voltage.

### Microstructure morphology and composition

#### Surface morphology

**Figure 2:**
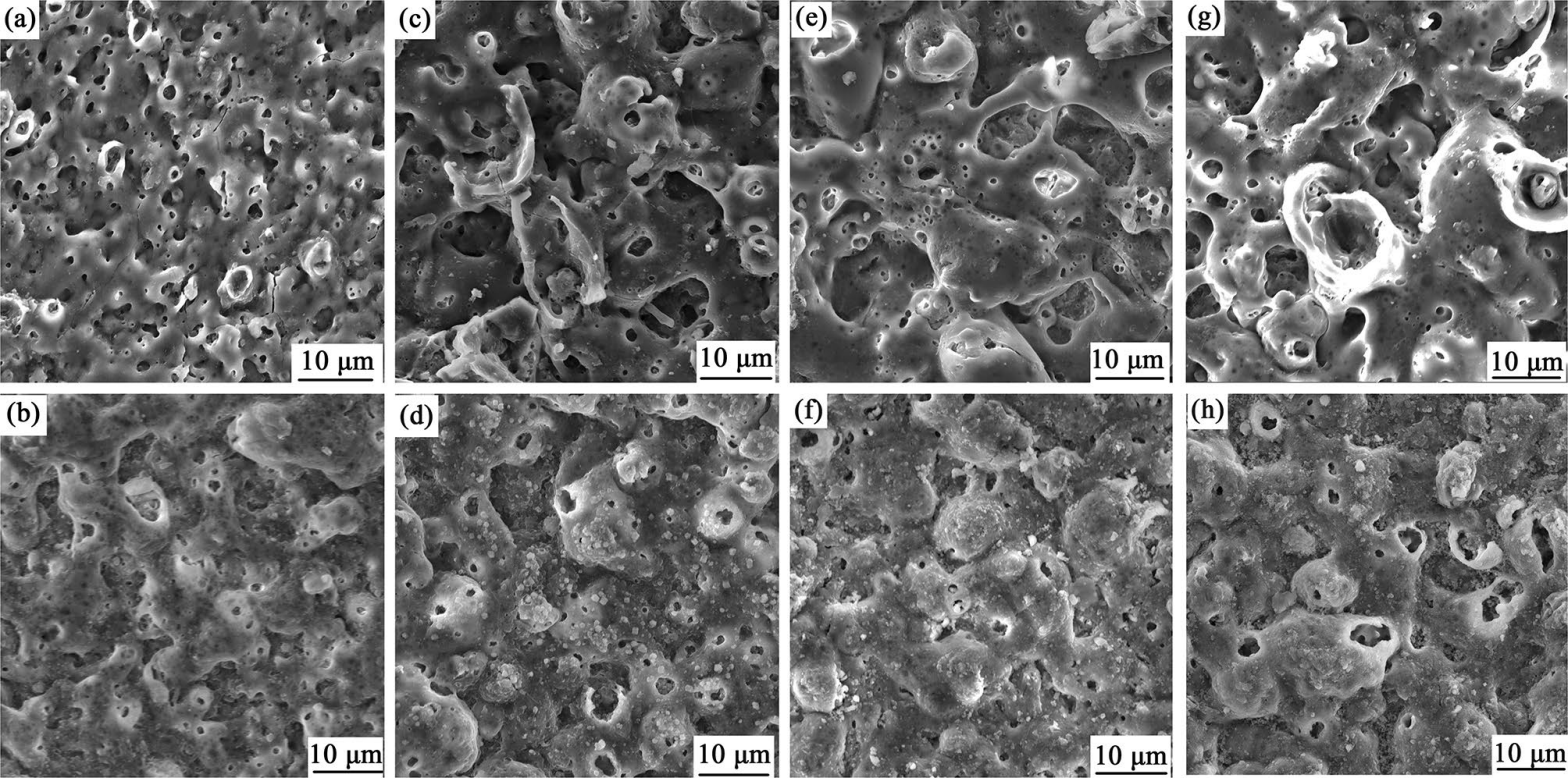
Surface morphology of the coatings. Subfigures (**a**),(**c**),(**e**) and (**g**) are the surface morphologies of the coatings without h-BN particles in the tenth, twentieth, thirtieth and fortieth minutes, respectively; Subfigures (**b**),(**d**),(**f**) and (**h**) are the surface morphologies of the coatings with h-BN particles in the tenth, twentieth, thirtieth and fortieth minutes, respectively.

Figure 2 shows the surface morphology of the coatings with and without h-BN particles at different PEO times. It can be seen from Fig. 2 that a large number of micropores appear on the coating surfaces, which is a typical characteristic of the PEO coating. But the difference due to the addition of h-BN particles can be clearly observed. Significantly, at the same oxidation time, the depth and size of the micropores on the coatings with h-BN particles (Fig. 2b,d,f and h) are smaller than that without h-BN particles (Fig. 2a,c,e and g). Some of the micropores are even closed due to the incorporation of h-BN particles, as shown in Fig. 2f. Furthermore, the depth and size of the micropores increase rapidly over time, while for the coating containing h-BN particles, the growth rate of the micropores is much smaller. This shows that the addition of h-BN particles has a great influence on the coating morphology.

**Figure 3:**
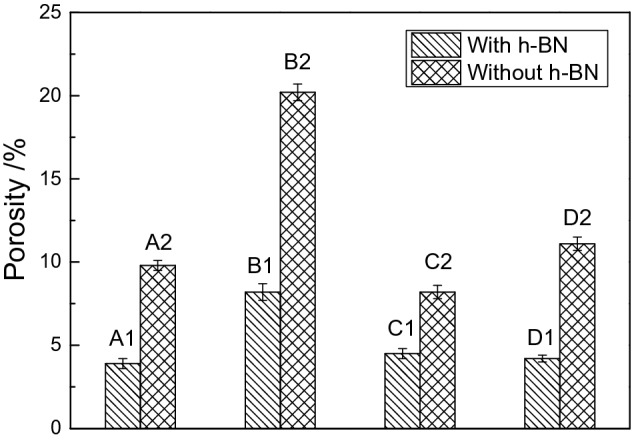
Porosity of the coatings with and without h-BN particles.

In the following, the pore characteristics of the coatings are further analyzed by software Image J. First, the scale in the software was set to be consistent with the SEM image. And then, the image was converted into 8 bit. The rectangle selection tool was used to select the area which need to be analyzed. After adjusting the threshold and measuring process, information such as the porosity and pore diameter of coating was automatically displayed. In order to improve the accuracy of the results, at least four different areas in the image were selected. Finally, the average porosity was obtained and shown in Fig. 3.

In the first twenty minutes, the porosity and maximum pore diameter of the coatings increase rapidly, especially for the coatings without h-BN particles. For the coatings without h-BN particles, the maximum porosity can reach 20.2%. This value is decreased by 59.4% compared to that of the coating with h-BN particles. Meanwhile, the maximum pore diameter is 10.2 μm for the coating with h-BN particles, which is 26.1% lower than that of the coating without particles. Next, the porosity and the maximum pore diameter of the coatings gradually decrease over time. At the end of the PEO treatment, the maximum porosity of the coatings with and without h-BN particles are 4.2% and 11.2%, respectively, indicating that the addition of h-BN particles remarkably improves the quality of coating. The result also corresponds with the morphologies observed in Fig. 2.

**Figure 4:**
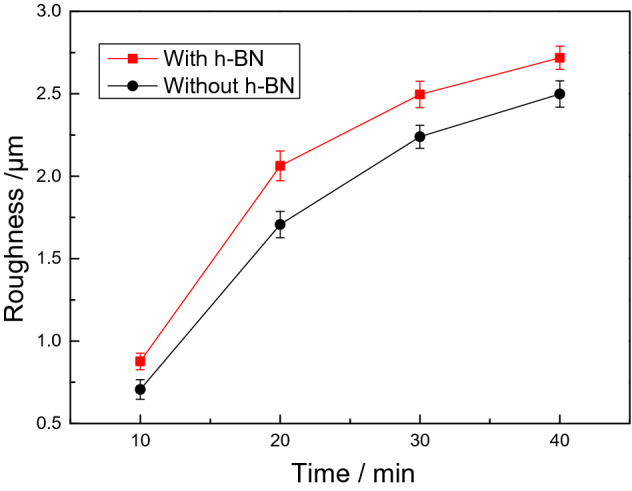
Surface roughness of the coatings at different oxidation time.

Surface roughness is another important parameter for evaluating coating quality. Figure 4 displays the surface roughness of the coatings as a function of time. It can be seen that, with the prolongation of the oxidation time, the surface roughness of the coatings increase continuously, but the growth rate of the surface roughness in the first twenty minutes is much higher than that in the subsequent time. This phenomenon may be related to the increasing degree of oxidation. During the initial stage of the PEO, the discharge on the surface changes from weak to strong, resulting in a rapid increase in the surface roughness. In the later stage, the conductivity of the coating deteriorates due to the changes in thickness, leading to the partial discharge that occurs on the part of the surface. Therefore, the rate of increase in protrusions and roughness on the coating become smaller. More importantly, the surface roughness of the coating with h-BN particles is generally higher than that of the coating without particles. For example, after 20 min of PEO treatment, the roughness of the coating is Ra 1.71 μm. This value is increased by 20.5% after the incorporation of h-BN particles. At the end of the PEO treatment, the surface roughness of the coating with h-BN particles, which rises to Ra 2.71 μm, is still 8.4% higher than that of the coating without particles. Therefore, the addition of h-BN particles into the electrolyte can not only reduce the porosity of the coating, but also slightly increase the surface roughness.

#### Thickness and cross-section microstructure of the coatings

Table 2 shows the thickness as well as the growth rate of the coatings with and without h-BN particles at different oxidation time. Here, the growth rate represents an increase in the thickness of the coating in every ten minutes intervals. Apparently, the thickness of the coatings increases with time, while their growth rate firstly increases and then decreases. By contrast, it can be seen that the addition of h-BN particles in the electrolyte can significantly promote the growth of the coating. For the coating without h-BN particles, the coating thickness is 42.01 μm at the end of the PEO treatment. Such thickness is increased by 42.05% when the h-BN particles are incorporated, with its value reaching 73.05 μm eventually. The growth rate of the coatings rises to its maximum in the twentieth minute for both samples. However, the maximum growth rate of the coating with h-BN particles is 3.08 μm/min, which is 58.25% higher than that of the coating without particles. The above phenomenon is caused by the changes of discharge intensity and discharge number in the PEO process.

**Table 2:** Thickness of the coatings at different oxidation time.

Time/(minute)	10	20	30	40
Sample	Without h-BN			
Thickness/(μm)	15.52	35.06	41.04	42.01
Growth rate/(μm/min)	1.55	1.95	0.60	0.10
Sample	With h-BN			
Thickness/(μm)	20.34	51.16	64.04	73.05
Growth rate/(μm/min)	2.03	3.08	1.29	0.90

In the first ten minutes, although the discharge intensity is relatively low after reaching the maximum breakdown voltage, the discharge number is relatively large. The duration time of the high current is relatively short and the average current value is 2.2 A. Therefore, the coating grows fast. In the second ten minutes, the discharge intensity reaches its maximum and the average current value is up to 3.1 A (as shown in Fig. 1). Thus the coating shows more higher growth rate than the first stage. However, the discharge number and intensity decrease rapidly as the thickness of coatings increase, resulting in a much lower growth rate of the coatings in the last twenty minutes.

**Figure 5:**
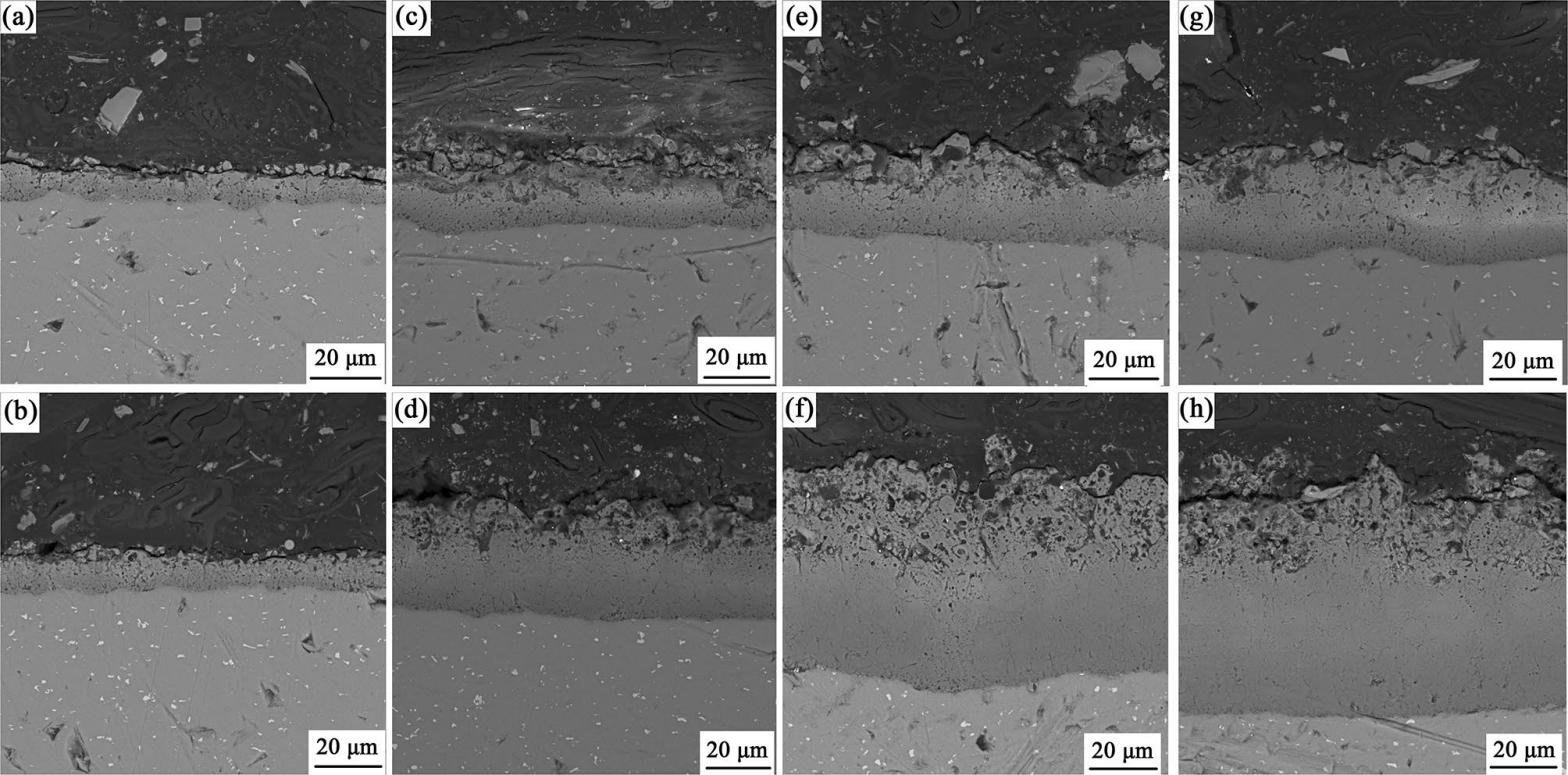
Cross-section morphology of the coatings. Subfigures (**a**),(**c**),(**e**) and (**g**) are the surfaces of the coating without h-BN particles in the tenth, twentieth, thirtieth and fortieth minutes, respectively; Subfigures (**b**),(**d**),(**f**) and (**h**) are the surfaces of the coating containing h-BN particles in the tenth, twentieth, thirtieth and fortieth minutes, respectively.

Moreover, the addition of h-BN particles also changes the cross-section morphology of the coatings. Figure 5 exhibits the backscattered electron (BSE) morphologies of the cross-section of coatings at different oxidation time. In the first ten minutes, both coatings are relatively loose with numerous micropores in the interior. With the increase of oxidation time, the proportion of dense layer increases rapidly and the growth of the coating is mainly manifested on the dense layer, especially for the coatings containing h-BN particles. It is also interesting to note that the incorporation of h-BN particles in the electrolyte may fill the discharge micropores in the coating, resulting in the reduction number of micropores in the dense layer.

#### Phase composition

**Figure 6:**
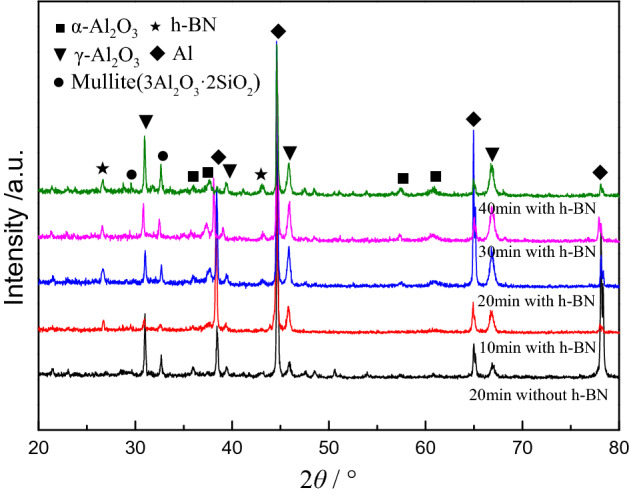
XRD analysis of the coatings.

To verify whether the h-BN particles enter the coating during the PEO process, the coating is analyzed by XRD. The results are shown in Fig. 6. The XRD pattern shows that the coating without h-BN particles in the twentieth minutes consists mainly of γ-Al_2_O_3_ and a small amount of α-Al_2_O_3_ and mullite. For the coating with h-BN particles at the same oxidation time, an h-BN diffraction peak corresponding to the 2θ angle of 26.5° appears, indicating that the particles in the electrolyte are inertly adsorbed or sintered to the PEO coating. In addition, the diffraction peaks of γ-Al_2_O_3_ at 46.5° and 67° increase and α-Al_2_O_3_ exhibits diffraction peaks at 37.5° and 62.3°. This indicates that the addition of h-BN particles not only promote the formation of γ-Al_2_O_3_, but also accelerate the conversion of γ-Al_2_O_3_ to α-Al_2_O_3_. It is just that the amount of conversion is less than the amount of formation due to different formation temperature. This may be related to the good thermal conductivity of h-BN particles. For the h-BN particles, as the oxidation time increases, the diffraction peak begins to appear at the 2θ angle of 43.7°. Since the maximum temperature of the discharge region can reach 5500 K^45^ during the formation of the coating, it can be considered that part of the h-BN lattice changes due to the high temperature caused by the PEO process. The change of h-BN lattice can in turn change its electrical conductivity^46^, resulting in a large current in the PEO process (as the stage B shown in Fig. 1), which promotes the PEO reaction. However, the diffraction peaks of the h-BN are relatively low throughout the whole PEO process, indicating that the amount of h-BN particles entering into the coating is very small. Nevertheless, it can still significantly promote the growth of the coating.

### Tribological properties

**Figure 7:**
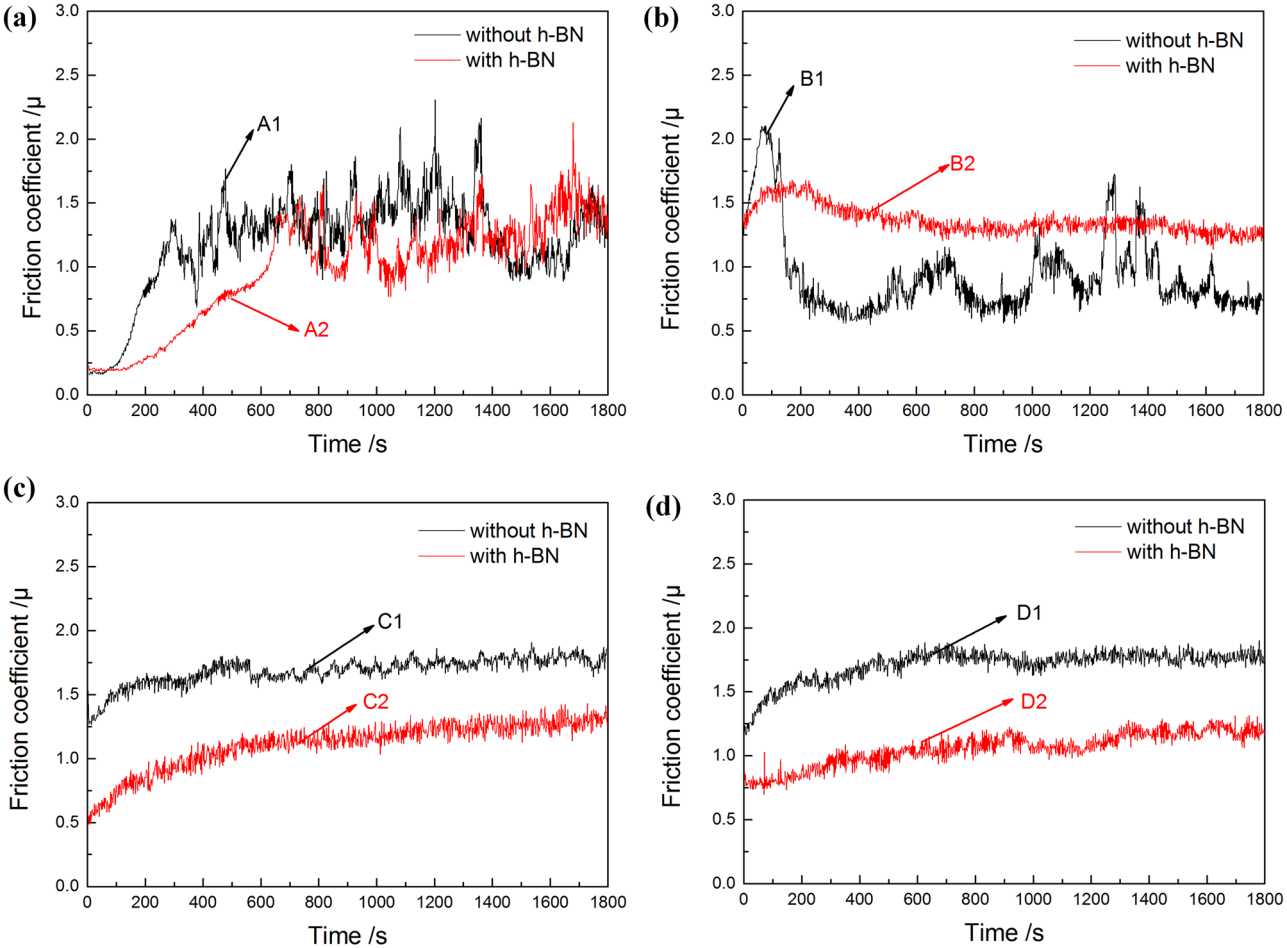
Variations of friction coefficient of the coatings during the tribological performance test. Subfigures (**a**),(**b**),(**c**) and (**d**) are the friction coefficient of the coatings that have been oxidized for 10 min, 20 min, 30 min and 40 min, respectively.

The friction coefficient of the coatings as a function of the test time is shown in Fig. 7. It can be seen that the initial friction coefficient has a certain relationship with the surface roughness. The initial friction coefficient of specimens with h-BN are generally less than that of without h-BN. For the coatings that has been oxidized for only 10 min, the friction coefficient increases significantly during the first one or two minutes of the test and then fluctuates around higher values (Fig. 7a). This indicates that the formed coatings on the substrate surface has worn out. As can be seen from Figs. 4 and 5, the roughness and thickness of the coatings are small, which may be the reason for the low initial friction coefficient and easy failure of the coatings. Moreover, the fluctuation of friction coefficient may due to the ploughing effect caused by the wear debris generated from the broken coatings.

When the oxidation time is extended to 20 min, the friction coefficient of the coatings with and without h-BN particles show different variation trends (Fig. 7b). The friction coefficient of coating without h-BN particles drops off quickly to a fluctuating state after a rapid increase in the first 20 s of the test, while the friction coefficient of the coating with h-BN particles gradually declines to 1.25 after a slight rise. The reasons might be attributed to the thickness of the coating with h-BN particles is higher than that of the coating without particles. Thus, the coating is not completely destroyed during the test, but its friction coefficient is still relatively high.

On the contrary, the values and the variation trends of the friction coefficient for the coatings that have been oxidized for more than 20 min are similar (Fig. 7c and d). Specifically, the friction coefficient increases slightly in the first 60 s of the test and then remains stable. When the PEO process comes to the end, the friction coefficient of the coating without h-BN particles is stabilized at 1.8 while the value decreases to 1.24 when the particles are added. This suggests that the addition of h-BN particles can effectively reduce the friction coefficient of the coating.

**Figure 8:**
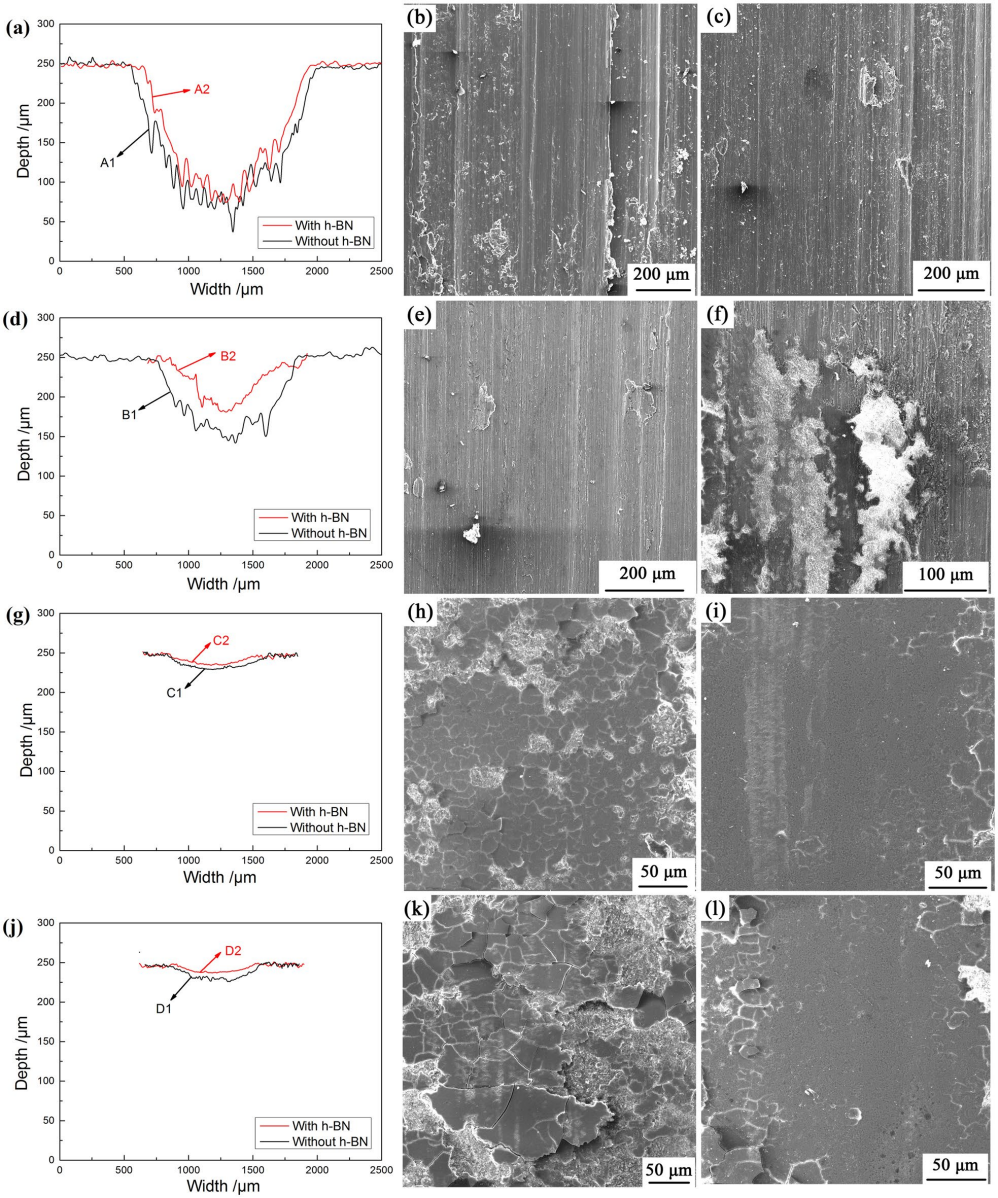
The 2D profiles and SEM of wear scar of the coatings. Subfigures (**a**),(**b**) and (**c**) are the wear scars and the SEM of the coatings that have been oxidized for 10 min; Subfigures (**d**),(**e**) and (**f**) are the wear scars and the SEM of the coatings that have been oxidized for 20 min; Subfigures (**g**),(**h**) and (**i**) are the wear scars and the SEM of the coatings that have been oxidized for 30 min; and Subfigures (**j**),(**k**) and (**l**) are the wear scars and the SEM of the coatings that have been oxidized for 40 min.

The 2D profiles as well as the SEM of the wear scar of the coatings are shown in Fig. 8, and the specific wear parameters, *i.e.* wear depth, wear width, wear volume and wear rate, are listed in Table 3. It can be seen that the wear scars of the coating A1 and the coating A2 have a typical U shape and the wear depths are large (Fig. 8a), which indicates that both coatings have severe wear and high wear rate. More specifically, the wear rates of the coatings A1 and A2 are 1.01 × 10^–3^ mm^3^/(N·m) and 0.83 × 10^–3^ mm^3^/(N·m), respectively. In addition, severe scratches and numerous wear debris are present on the bottom surface of the wear scar, as shown in Fig. 8b and c. This means that abrasive wear occurs during the rubbing, which is also consistent with the fluctuation of the friction coefficient in Fig. 7a. It can be considered that the point contact occurs between the high hardness ceramic ball and the coating at the initial presence of friction, leading to high shear stress during the reciprocating process. Wear debris will be generated under the repeated action of high shear stress, and abrasive wear happens between the coating and the ball, causing the failure of the coating. For this situation, the effect of h-BN particles in the coating is not obvious. Similarly, the profiles of the wear scar of the coating B1 and coating B2 also have a typical U shape (Fig. 8d). However, the amount of grooves and wear debris at the bottom of the wear scars have dropped a lot, and the wear rates have decreased to 0.44 × 10^–3^ mm^3^/(N·m) and 0.15 × 10^–3^ mm^3^/(N·m), respectively.

**Table 3:** Parameters of the wear scars.

Samples	Depth/(μm)	Width/(mm)	Wear volume/(mm^3^)	Wear rate/(mm^3^·N^-1^·m^-1^)
A1	202.75	1.41	96.57 × 10^–2^	1.01 × 10^–3^
A2	177.05	1.33	79.73 × 10^–2^	0.83 × 10^–3^
B1	110.04	1.14	42.08 × 10^–2^	0.44 × 10^–3^
B2	49.63	0.85	14.08 × 10^–2^	0.15 × 10^–3^
C1	22.56	0.81	6.03 × 10^–2^	0.06 × 10^–3^
C2	14.75	0.59	2.89 × 10^–2^	0.03 × 10^–3^
D1	34.72	0.82	9.47 × 10^–2^	0.10 × 10^–3^
D2	23.67	0.63	4.94 × 10^–2^	0.05 × 10^–3^

The wear resistance of the coatings C1, C2, D1 and D2 have greatly improved. It can be seen from Fig. 8g and j that the wear scars are arc-shaped, indicating that surface contact happens between the coating and the ceramic ball. Apparently, this leads to shallow wear depths and smooth bottoms, with their wear rates reducing to 0.06 × 10^–3^ mm^3^/(N·m), 0.03 × 10^–3^ mm^3^/(N·m), 0.10 × 10^–3^ mm^3^/(N·m) and 0.05 × 10^–3^ mm^3^/(N·m), respectively. Furthermore, the coatings C1, C2, D1 and D2 are not damaged and the surfaces of the wear scars are smooth during the test. This may be due to an increases in the content of γ-Al_2_O_3_ and α-Al_2_O_3_ with higher hardness in the coating (as shown in Fig. 6), which has a higher hardness. On the other hand, some large uniform size lamellar accumulation appears on the wear scars of coatings C1 and D1, while the bottom surface of the wear scars of coatings C2 and D2 are more flatter and have less lamellar accumulation that exists only at the edge of the wear scars, as shown in Fig. 8h,i,k and l. The formation of lamellar accumulation may due to the loose layer which has high hardness, low toughness and low fatigue wear that can be sheared and pressed into lamellar accumulation because of the excessive hardness of the ceramic ball^39^. The flatter bottom surface of coatings C2 and D2 may relate to the presence of h-BN particles in the coating. The h-BN particles are referred to as “white graphite”, which means that it has good self-lubricating property and can also reduce the frictional shear stress between the coating and the Si_3_N_4_ ball. These advantageous properties will lead to the smaller wear scar and wear rate, thereby further proves that the composite ceramic coating has better tribological properties.

**Figure 9:**
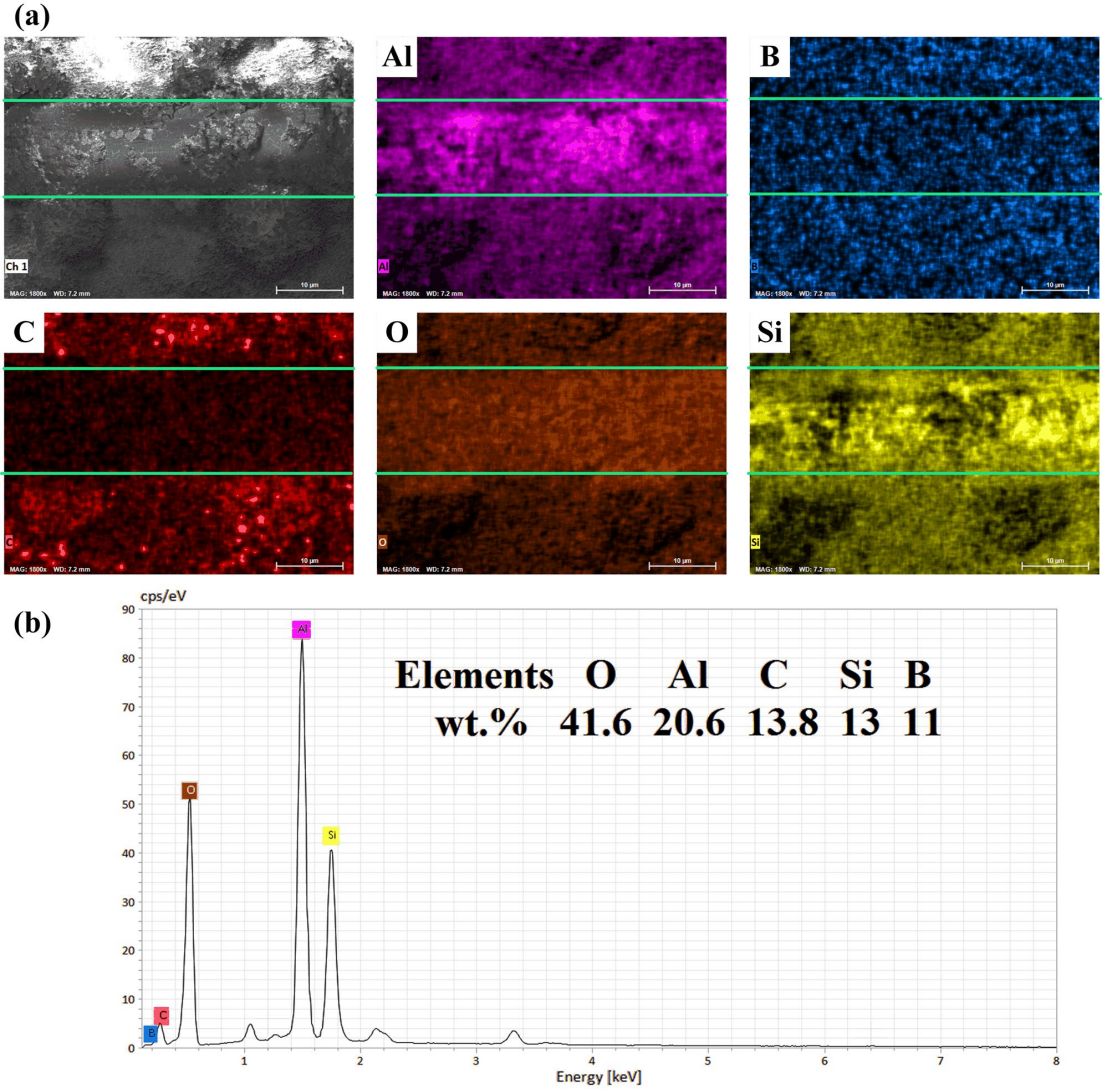
EDS of the wear scars. Subfigure (**a**) and (**b**) are the EDS of the wear scar surfaces of the coatings with h-BN particles that have been oxidized for 30 min, respectively.

Besides that, the EDS results of the coating C2 after wear test, as displayed in Fig. 9, also show that, due to the addition of h-BN particles, the B elements is evenly distributed on the scratch and the surface with a content of about 11 wt.%. It means that the h-BN particles have been deposited into the coating through the PEO process. Furthermore, the aggregation of the Al, O and Si elements are also present on the scratch area. The Al element may come from the matrix, and the Si element may come from the counter ball.

### Deposition mechanism

**Figure 10:**
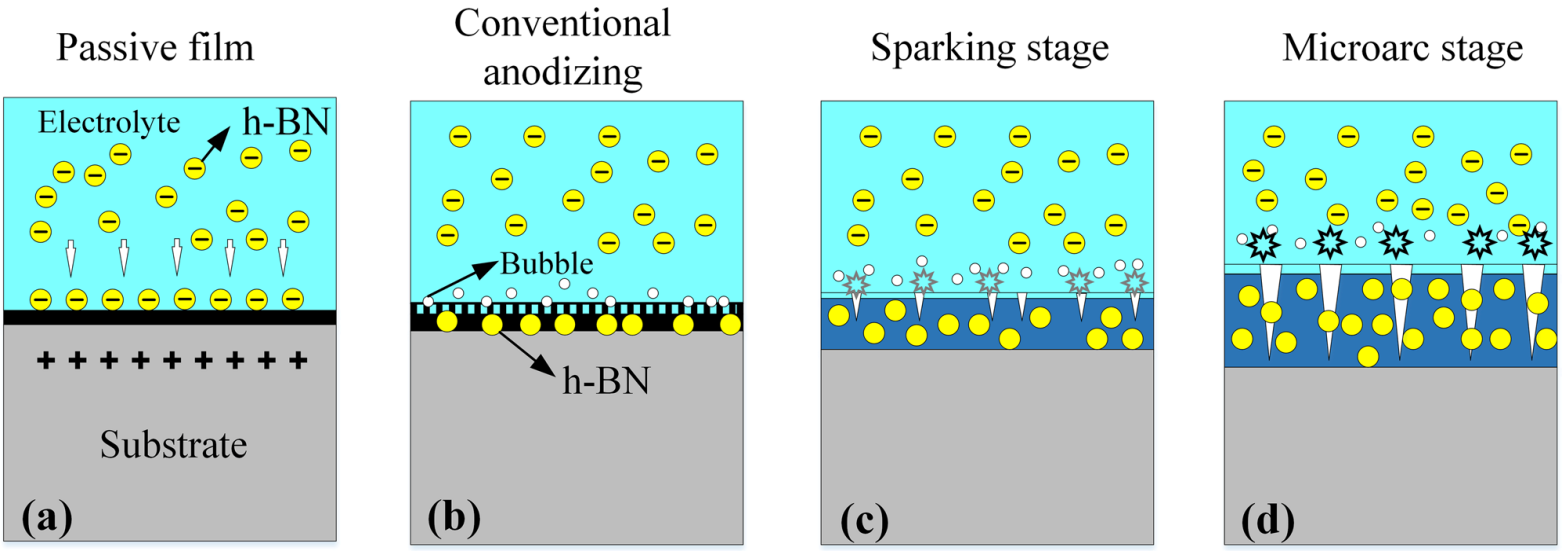
Deposition mechanism of h-BN particles.

Figure 10 is the schematic diagram of the deposition mechanism of h-BN particles. The PEO process with h-BN particles in the electrolyte can be divided into four stages according to the changes of current. The first stage is the formation of passivation film. At this stage, with the increase of supply voltage, the current begins to rise gradually (stage 1 as shown in Fig. 1) and the heat dissipates, resulting in a passivation of the sample surface. Meanwhile, part of the negatively charged modified h-BN particles aggregate towards the surface of the sample under the action of electrophoretic force.

As the supply voltage and heat dissipation increase, anodization occurs. The h-BN particles accumulated on the surface are adsorbed by the sample to form a composite oxidation film. At the same time, the electrolyte is electrolyzed and bubbles begin to appear on the surface. The bubbles generated on the surface will isolate the sample from direct contact with the electrolyte, resulting in a drop in current (stage 2 as shown in Fig. 1). When the supply voltage reaches the breakdown voltage, the oxidation film is destroyed by impact or tunnel ionization and small illuminating spark are observed on the sample surface. At this point, the PEO process enters the third stage, which is known as the spark discharge stage. It is interesting to note that the current begins to rise slowly again in this stage (stage 3 as shown in Fig. 1). One of the reason may be that a rapid increase in spark intensity leads to a decrease in the number of bubbles^47^. Another reason may be that some originally interconnected small passages form larger discharge passages due to the increase of park intensity. In addition, the increase of heat dissipation causes the original oxidation film to be broken, melted and sprayed outward. The molten material wraps the h-BN particles and condenses in the coating to form a composite coating. In the last stage, uniform white light firstly appears on the surface of the sample, which means that continuous discharge breakdown occurs. Therefore, the coating grows rapidly, and the current decreases correspondingly until a stable value is reached (stage 4 as shown in Fig. 1). The h-BN particles in the electrolyte also quickly enter the coating or the discharge channels simultaneously, such that more particles are observed in the molten protrusions and micro-pores on the surface of the coating (the white spot on the surface as shown in Fig. 2d). Then, the uniform white light gradually turns into a plurality of individual yellow sparks and the growth rate of the coating is reduced. As a result, less particles are observed on the coating surface as the oxidation time increases, as shown in Fig. 2f and h.

## Conclusion

The h-BN particles that have been modified by polyethylene glycol were added into the PEO electrolyte, and the composite ceramic coating was prepared on the surface of ZL109 alloy. The growth regularity and tribological behavior of the composite ceramic coating were analyzed. The following conclusions are obtained:

(1) The h-BN particles in the electrolyte are inertly incorporated into the coating. As a result, the porosity of the coating is reduced, and the thickness as well as the roughness of the coating are increased.

(2) By decreasing the oxidation current in the early stage and increasing the oxidation current in the later stage of the PEO process, the introduction of h-BN particles in the electrolyte can increase the growth rate of the coating and accelerate the conversion of γ-Al_2_O_3_ and α-Al_2_O_3_ in the coating.

(3) Compared to the coating without h-BN particles, the composite ceramic coating containing h-BN particles has a lower friction coefficient and a lower wear rate under dry sliding condition. The composite ceramic coating that have been oxidized for 30 min has the lowest wear rate.
